# Fundamental role of arsenic flux in nanohole formation by Ga droplet etching on GaAs(001)

**DOI:** 10.1186/1556-276X-9-309

**Published:** 2014-06-18

**Authors:** David Fuster, Yolanda González, Luisa González

**Affiliations:** 1 Instituto de Microelectrónica de Madrid (IMM-CNM, CSIC), Isaac Newton 8, Tres Cantos, Madrid 28760, Spain

**Keywords:** Droplet epitaxy, Quantum dots, Nanopatterning

## Abstract

Nanoholes with a depth in the range of tens of nanometers can be formed on GaAs(001) surfaces at a temperature of 500°C by local etching after Ga droplet formation. In this work, we demonstrate that the local etching or nanodrilling process starts when the Ga droplets are exposed to arsenic. The essential role of arsenic in nanohole formation is demonstrated sequentially, from the initial Ga droplets to the final stage consisting of nanoholes surrounded by ringlike structures at the surface and Ga droplets consumed. The kinetics of local etching depends on the arsenic flux intensity, while the ringlike structures are basically the same as those formed underneath the droplets in the absence of arsenic. These structures show motifs with well-defined crystalline facets that correspond to those expected from surface energy minimization. These experimental results are qualitatively analyzed for a better understanding of the nanohole formation underlying processes.

## Background

Semiconductor quantum dots (QDs) have been extensively studied in the last years. The quantum confinement effect of these structures allows the design of novel devices related to a wide range of applications in electronics and optoelectronics [1,2]. Self-assembled QDs have been successfully fabricated by the epitaxial growth of a layer in a lattice-mismatched III-V semiconductor system through the well-established Stranski-Krastanov (SK) process. Although a lot of fundamental physical understanding and a variety of applications have been realized using this kind of QDs, custom design of the shape and size of the nanostructures is seriously constrained by the self-assembling processes. The droplet epitaxy (DE) technique is another way to obtain QDs with some advantages over the SK mode [3]. For example, QDs of lattice-matched materials (as GaAs/AlGaAs) can be formed by DE. A variety of shapes have been obtained by this technique: dots, rings, concentric double-ring structures, dot pairs [4-6]. Several nanostructures fabricated by DE have been implemented in devices as lasers, detectors, single-photon emitters, and solar cells [7-11]. An effect of the DE technique is the ‘nanodrilling’ of the substrate or local etching by the droplets, which has been recently studied as an *in situ* procedure to obtain nanoholes [12-14]. The nanodrilling process has its origin in the etching of a semiconductor by a liquid metal [15-17]. For Ga droplets on GaAs(001), we have observed the etching process for substrate temperatures ≥450°C. The nanoholes formed by DE provide cleaner interfaces than those formed by any other *ex situ* lithographic techniques without any need of special treatments for further regrowth processes. By depositing a III-V semiconductor of lower bandgap, the nanoholes can be refilled and QDs are formed at the nanoholes. The density of the holes determines the density of the QDs and their size depends on the amount of deposited material to form them, being relatively easy to tune the emission wavelength independently of the density [18]. The optical properties of these QDs are also influenced by the characteristics of the nanoholes. For example, the depth and shape of the nanoholes are determinant in obtaining GaAs/AlGaAs QDs with narrow line shape and null fine structure splitting [19]. Moreover, the kind of QD/nanohole interface would be in the origin of the charge exciton species predominant in the micro-PL spectra of InAs/GaAs QD [13] and in the formation of QD molecules instead of single QD [20]. In order to take advantage of all the potential of droplet epitaxy as a nanopatterning technique, a complete understanding of the mechanisms of nanohole formation is mandatory.

A lot of experimental and theoretical work has been reported ([21], Chap. 3 and references therein, [22,23]) to explain the droplet crystallization evolution at a low temperature (<300°C, where nanoholes are not observed). Although some works have also been dedicated to model local droplet etching [24,25], experimental results showing step by step the full process would be of great help for a deeper understanding.

In this work, we monitor the hole formation process during the transformation of Ga droplets into nanoholes on GaAs(001) surfaces at substrate temperature *T*_S_ = 500°C. This process takes place when Ga droplets are exposed to arsenic. The essential role of arsenic in nanohole formation is demonstrated sequentially, from the initial Ga droplets to the final stage consisting of nanoholes at the surface and Ga droplets completely consumed. For this purpose, we have grown samples at different stages of the local etching process under several annealing conditions, and we have studied the dependence of the depth of the nanoholes with arsenic flux and annealing time. The experimental results are qualitatively analyzed for a better understanding of the processes underlying the nanohole formation.

## Methods

The samples under study were grown on GaAs(001) substrates by molecular beam epitaxy (MBE) in two different reactors: a homemade MBE system and a RIBER (Paris, France) Compact 21E MBE system. Previous to any growth process, oxide from the GaAs(001) surface was thermally removed and a 150-nm-thick GaAs buffer layer was grown at a growth rate of 0.5 monolayer (ML) per second at substrate temperature *T*_S_ = 580°C. The droplets were formed by depositing at *T*_S_ = 500°C 4 ML of Ga at 0.04 ML/s, denoted in equivalent monolayers of GaAs on GaAs(001). For ensuring a minimal As background pressure in the MBE reactor before Ga is deposited, we follow specific procedures in the different MBE systems. In the RIBER Compact 21E MBE, once the As cell valve is closed, we wait until the background pressure reading is lower than 3 × 10^−9^ Torr. In the homemade MBE system, we need to cool down the As cell besides closing its valve, to achieve a final background pressure reading lower than 1 × 10^−9^ Torr. With these procedures, reproducible results are obtained independently on the system where the samples were grown. After droplet formation, the surface was annealed either under As_4_ flux or in the absence of arsenic during different times. The different As fluxes used in this work are also indicated in equivalent ML/s, 1.40, 0.70, and 0.08 ML/s, and were measured by monitoring the specular beam RHEED oscillations during GaAs growth limited by V element [26]. The samples annealed under arsenic flux were cooled down in the presence of arsenic before taken out from the MBE chamber.

The morphology of Ga droplets and nanoholes was measured by atomic force microscopy (AFM) in a Nanotec (Tres Cantos, Spain) and/or a Veeco Dimension Icon (Plainview, NY, USA) scanning probe microscopy system, using Nanosensors silicon cantilevers (*K* = 40 to 50 N/m, Neuchatel, Switzerland) with small radius tips (≤7 nm) in tapping mode. For AFM data analysis, the free Gwyddion software was employed.

## Results and discussion

Contrary to the previously published works [12-14], our results show that in the absence of arsenic, the Ga droplets formed at *T*_S_ = 500°C remain at the GaAs(001) surface after growth interruptions (at *T*_S_ = 500°C) ranging from 5 to 30 min. Under these experimental conditions, no nanoholes appear across the surface. An actual low As pressure in the system background is the key point for reproducing this result. In fact, in our homemade MBE system, nanoholes appear (results not shown) if the As cell is not cooled down, besides being fully closed, previously to Ga deposition for droplet formation, in complete agreement with the experimental results reported by other authors up to date.

**Figure 1:**
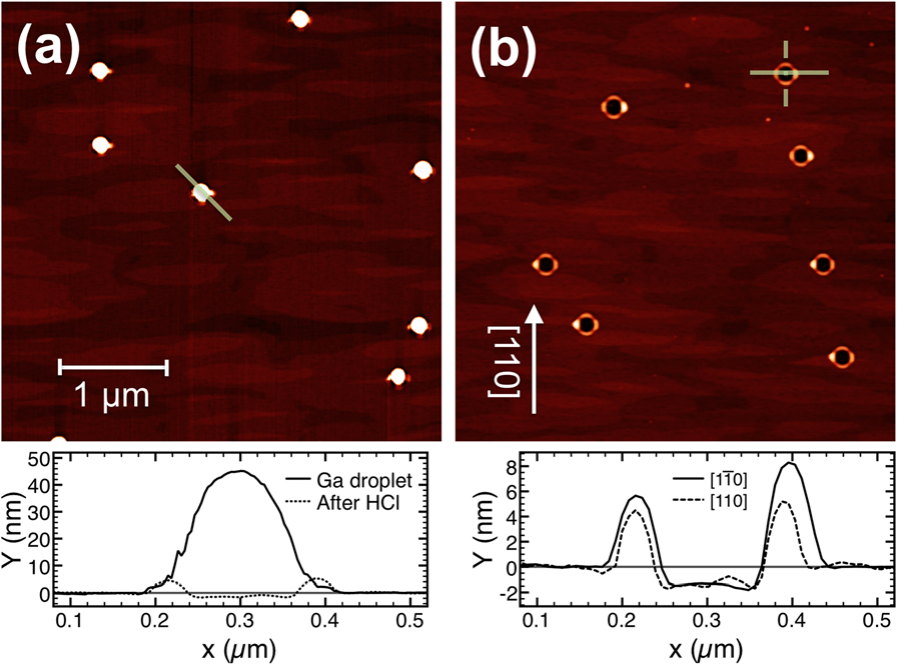
**AFM images of Ga droplets. (a)** 4 × 4 μm^2^ AFM image of Ga droplets formed on the GaAs(001) surface at substrate temperature *T*_S_ = 500°C after a growth interruption of 30 min; the profile plotted below corresponds to the line crossing a Ga droplet in the AFM image. The dotted line represents the depression measured underneath the Ga droplet after HCl etching. **(b)** 4 × 4 μm^2^ AFM image of the sample of Figure 1a after removal of Ga droplets by HCl etching. The profiles along the two directions marked on the image are shown below.

For the growth parameters used in this work, the obtained Ga droplets are typically 45 nm high and 120 nm full width at half maximum (FWHM) with a density of 4.5 × 10^7^ cm^−2^ (Figure 1a). The size and density of the Ga droplets are the same as those in a sample with 30 min of growth interruption at *T*_S_ = 500°C and in a sample that has immediately been cooled down after Ga deposition (not shown). This indicates that for the low Ga growth rate employed in this work (0.04 ML/s, the Ga cell is opened 100 s for depositing 4 ML), the droplet ripening, observed after Ga deposition by other authors [12,25], takes place in our case simultaneously to Ga deposition. Here, we want to point out that the deposition rate used in the previously cited published works was 1 ML/s, so the Ga deposition time lasts for only a few seconds and the ripening process that happens during the annealing time can be detected by AFM characterization after growth.

When the Ga droplets are removed by HCl chemical etching (Figure 1b), the surface shows ≈ 2-nm-deep flat depressions in the areas previously occupied by the droplets. These depressions are caused by the dissolution of the GaAs substrate by metallic Ga droplets, incorporating As atoms from the substrate until a stable composition is reached. The composition of the resulting alloy is limited by the arsenic solubility in Ga at 500°C [16], being Ga-rich enough to be etched by HCl. The observed depressions are surrounded by GaAs ringlike structures whose diameter is similar to that of the corresponding Ga droplet. A similar phenomenology was observed in Ga droplets formed at *T*_S_ = 350°C [6] and in ten times larger Ga droplets created by annealing a GaAs(001) substrate at 670°C, above the surface congruent evaporation temperature [27].

These depressions show a quasi-square shape with their sides along <110 > directions. They are surrounded by GaAs ringlike structures with four sectors (one for each side of the depression) aligned along <110 > directions. Among the four sectors of the ring, three are similar in height (≈5 nm). The other one is higher (≈8 nm) and always appear along one of the [110] sides; from this point on, this sector will be referred as the main sector.

**Figure 2:**
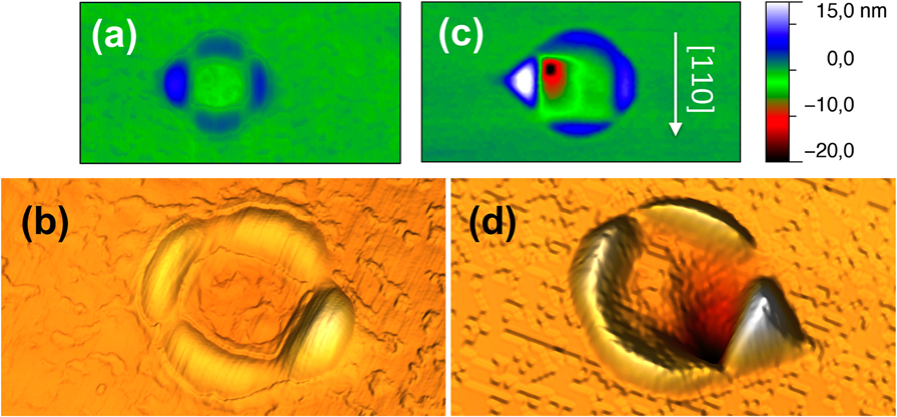
**AFM images of ringlike structures before and after As exposure of Ga droplets. (a)** 600 × 300 nm^2^ AFM image of the ring structure, formed at a substrate temperature of 500°C, remaining after the Ga droplet was removed by HCl. **(b)** 3D representation of the ring structure. **(c)** 600 × 300 nm^2^ AFM image of the ring structure and nanohole obtained after annealing the Ga droplet under an As flux of 0.70 ML/s for 30 s. **(d)** 3D image of the same structure where the facets of the highest structure (main sector) surrounding the ring are clearly seen.

**Figure 3:**
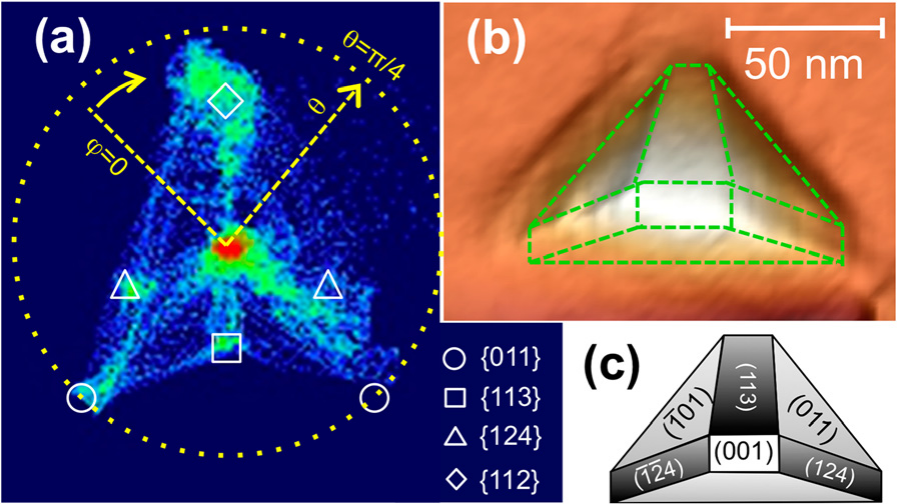
**Calculated surface orientation map, 3D planar view representation, and scheme of the main sector structure. (a)** Calculated surface orientation map from the AFM image of the main sector similar to that shown in Figure 2d. The arrows indicate the increasing direction of the polar coordinates (*θ*, *φ*) of the [*hkl*] vectors. Empty symbols mark the family planes present. **(b)** 3D planar view representation of the AFM image where the facet edges have been highlighted by dashed lines. **(c)** Scheme of the main sector structure obtained from the surface orientation map with the facet indexing corresponding to the different family planes.

The long-time stability of the Ga droplets can be drastically interrupted in the presence of arsenic. In Figure 2, we show a detailed AFM characterization of the kind of nanostructures that are formed without (a, b) and with (c, d) As irradiation of a Ga droplet. As fundamental differences, we observe that the Ga droplet have disappeared and the flat square-shaped depression inside the rings, observable after chemical etching of the Ga droplets (Figure 2a,b), has evolved in the presence of arsenic towards a deep and narrow hole, which is systematically located at one of the two corners adjacent to the main sector of the surrounding ring. We also observe that after arsenic exposure, the main sector increases in height (8 to 15 nm) and shows better defined crystalline facets. The facet orientation can be determined by high-resolution AFM measurements. Here, we want to notice that the fidelity of AFM imaging of nanostructures decreases with increasing slope of the sidewall facets due to the limitations in feedback gain and distortions caused by the tip-sample convolution. Moreover, the small area size of the main sector facets in comparison with the tip radius (≤7 nm) limits the number of experimental points to be used for facet {*hkl*} indexing. Figure 3a presents the surface orientation map obtained from the AFM image shown in Figure 3b. These maps are obtained by calculating the normal vector for each image point using the nearest-neighboring image points [28,29]. Each normal vector is determined by the polar coordinates (*θ*, *φ*) of the [*hkl*] vectors, where *θ* is the inclination angle between [*hkl*] and the [001] substrate normal and *φ* denotes the in-plane azimuth angle of the [*hkl*] vector with respect to the [100] substrate direction. Besides all the experimental constraints, zones with accumulation of points clearly appear in Figure 3a. The polar coordinates of these point accumulation zones can be assigned to several families of planes: {011}, {113}, {124}, and {112} (indicated in the map by circle, square, triangle, and diamond symbols, respectively). The brightest spot at the center (not labeled) corresponds to the (001) surface plane. Although our experimental results point out that the steep wall close to the deep hole would be indexed as {112}, the experimental constraints (AFM tip geometry and main sector size) could distort the experimental measurements and the true facet would be steeper than {112}.

Figure 3b shows a plane view of a three-dimensional (3D) representation of an AFM image with the edges marked by dashed lines to clearly distinguish the facets; a scheme of the proposed structure with the corresponding facets labeled is depicted on Figure 3c. The facets forming the main sector correspond to the family planes that are obtained by surface energy minimization calculations [30-32] for the equilibrium shape of GaAs crystals. So, we can conclude that this faceted structure is a minimum energy state of the GaAs grown from Ga coming from the droplet and As coming from the substrate (in the absence of arsenic) and also from the incoming arsenic flux when the As cell valve is opened.

The above described results point out the similarities of the nanorings formed at the surface when the Ga droplets are exposed to arsenic and below the Ga droplets in the absence of arsenic. But there is a fundamental difference between both results: nanoholes only appear if the droplets are exposed to arsenic.

**Figure 4:**
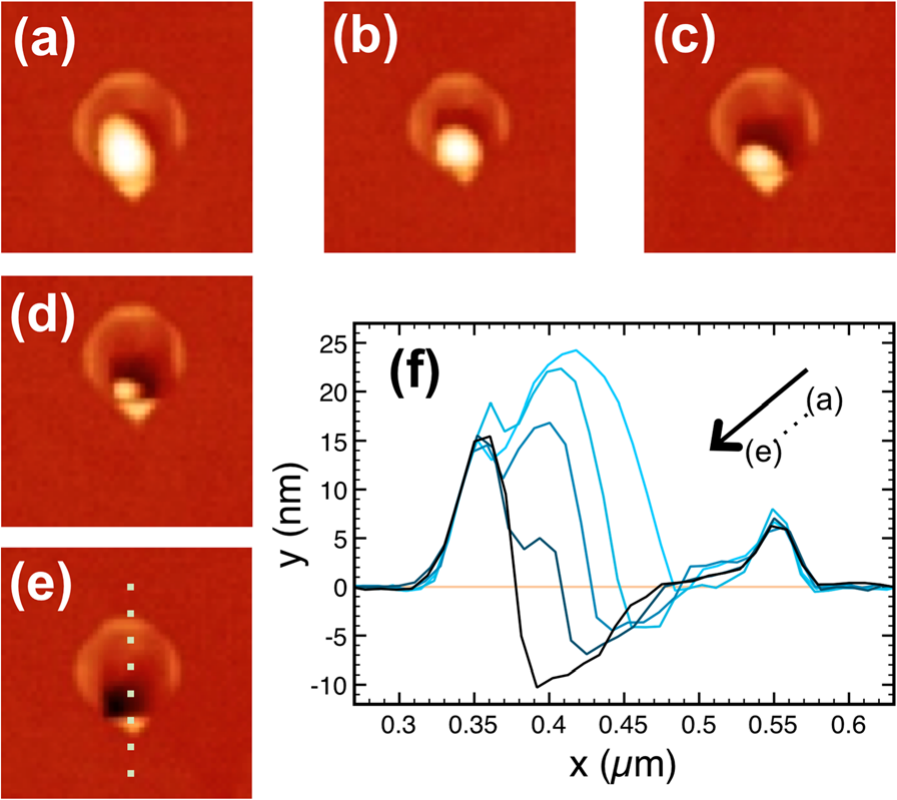
**AFM images of different stages of the nanodrilling process and profiles along the**$\left[1\bar{1}0\right]$**direction. (a-e)** 500 × 500 nm^2^ AFM images of different stages of the nanodrilling process during the Ga droplet consumption. **(f)** Profiles along the $\left[1\bar{1}0\right]$ direction [dashed line marked in (e)], normalized to the smallest ring diameter, showing the progressive droplet reduction, the local etching of the GaAs substrate, and the progressive filling of the part of the hole free of Ga droplet.

Considering the decisive role of arsenic in nanodrilling, it would be expected that the rate of this process will directly depend on the supplied As flux. At low As flux, it has been possible to capture different stages of the droplet evolution. In Figure 4, we show AFM images of the evolution of Ga droplets when exposed at a low As flux (0.08 ML/s) at *T*_S_ = 500°C. It can be clearly observed how the size of the Ga droplet progressively decreases. The reduced droplet remains always situated at one of the two corners of the main sector. The sequence starts with a 25-nm-high Ga droplet (Figure 4a), already smaller than the original Ga droplet before arsenic exposure, which progressively decreases in size (Figure 4b,c,d) until the total consumption (Figure 4e). The profiles extracted in each stage along the $\left[1\bar{1}0\right]$ direction (dashed line marked in Figure 4e) are shown in Figure 4f. We observe an increase of the depth of the hole synchronized with the droplet consumption. Simultaneously, in the opposite side to the location of the remaining droplet (right-hand side in the profiles), we can observe the progressive filling of the part of the hole that is not already covered by the Ga droplet. This fact could be related to the definition of B-type facets inside the nanodrilled holes that, under certain growth conditions, preferentially incorporate Ga with respect to (001) surfaces [33]. The Ga atoms incorporated at B-type walls would come from the Ga droplet and/or from the surface Ga atoms during the crystallization process. Both the etching process and the growth of GaAs from Ga coming from the droplets are resumed when the droplet ends, with the final result of a nanohole surrounded by GaAs ringlike structures. The presence of droplets attached to one corner of the ringlike structures strongly resembles, at another size scale, to those results obtained in Ga droplets of approximately 2-μm diameter produced at substrate temperatures above the congruence evaporation point [34].

These results show that the nanohole formation process is activated when Ga droplets are exposed to arsenic, while in the absence of arsenic, only flat depressions beneath the Ga droplets are observed. Arsenic exposure also leads to the consumption of the Ga droplets.

It is well known that As supply to Ga droplets triggers the onset of different processes [4,21-23], in particular a change in Ga droplet composition due to the incoming arsenic diffusion through metallic Ga, driving the Ga droplet arsenic content out of the equilibrium value at the corresponding temperature. In order to restore the arsenic equilibrium composition, Ga atoms belonging to the substrate would migrate towards the Ga droplet, if kinetics is not inhibited, with the subsequent enhancing of local substrate dissolution and the onset of the nanohole formation process. This explains why nanoholes penetrating in the substrate only appear in the presence of arsenic at high enough substrate temperatures. Simultaneously to the nanodrilling effect, GaAs is forming around and at the edge of the Ga droplet as has been previously reported [6,23], leading to its consumption at a rate that will depend on *T*_S_ and As flux. In this way, there is a competition between Ga coming from the substrate that incorporates at the Ga droplet and droplet consumption by forming GaAs. Altogether, a Ga droplet under As gives rise to ringlike nanostructures surrounding a deep and narrow hole that can penetrate up to tens of nanometers into the substrate.

These processes are closely related to the Ga-assisted vapor-liquid-solid growth of nanowires, where the incorporation of Ga and As and the GaAs crystallization take place below and around the Ga droplet [35], being in our case the source of Ga is the GaAs substrate instead of an incoming Ga flux.

**Figure 5:**
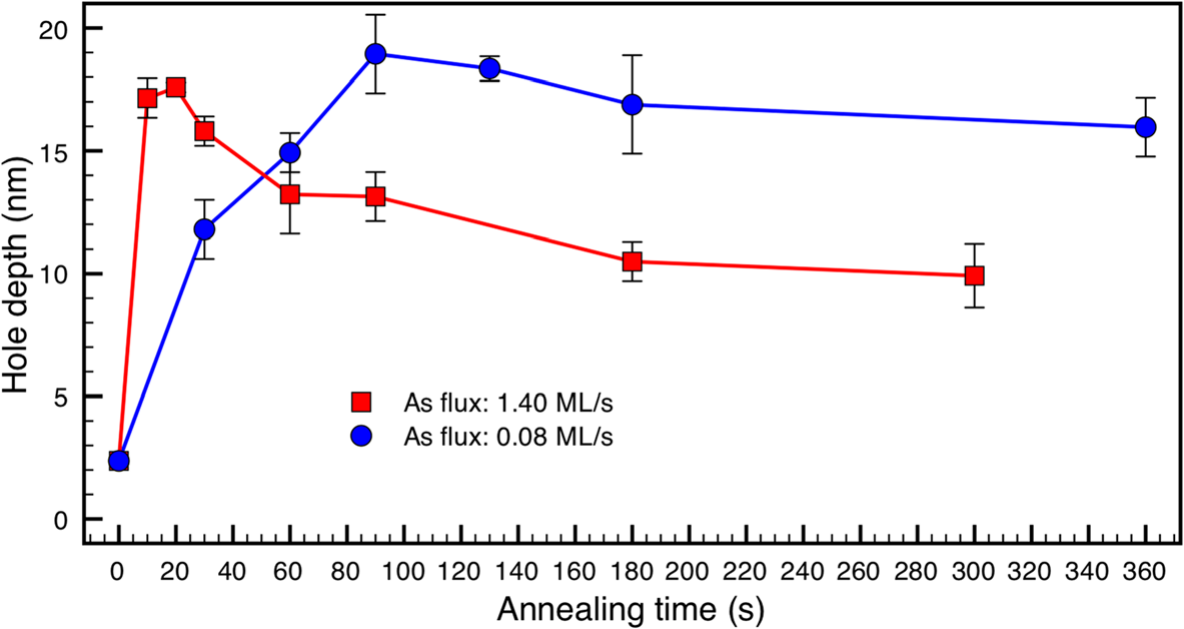
**Hole depth as a function of the annealing time of Ga droplets.** Under two different arsenic fluxes (0.08 and 1.40 ML/s) at constant substrate temperature *T*_S_ = 500°C.

According to the critical role of arsenic in nanohole formation, arsenic flux and time to arsenic exposure of Ga droplets would be key parameters to control the process. In order to have a deeper insight into this process, samples exposed to different As flux intensities during different annealing times, keeping the substrate temperature at *T*_S_ = 500°C, were grown and characterized.Figure 5 shows the average depth of nanoholes as a function of annealing time for the two different As flux intensities employed. The data points at annealing time 0 s correspond to the depth of the depressions remaining after HCl etching of the Ga droplets annealed in the absence of As. On the one hand, it is observed that the depth of the nanoholes depends on arsenic flux and time duration to arsenic exposure with two characteristic rates, faster and slower, before and reaching the maximum depth, respectively. On the other hand, the maximum nanohole depth is achieved at a longer annealing time for a lower As flux. Moreover, once the nanohole maximum depth has been achieved, a further annealing time under As flux leads to a reduction of the nanohole depth.

In view of our results, we can outline the following processes running during the annealing of Ga droplets under As exposure, which are associated to the characteristic evolution rates: local etching by the metallic Ga droplets (I) active until the Ga droplets are consumed by GaAs growth (II) and evolution of nanoholes to shallower structures (III).

In this context, it can be explained that the annealing time for reaching the nanohole maximum depth by nanodrilling beneath the Ga droplet (process I) depends on As flux, as the consumption rate of the droplet by GaAs formation (process II) depends on As flux in MBE growth under growth conditions limited by V element [26]. Once the etching is over by consumption of the Ga droplets (nanohole maximum depth achieved), a further annealing time under As flux leads to a reduction of the nanohole depth due to the incorporation of Ga atoms at B-type walls coming from the lateral movement of Ga surface atoms during the annealing process, a behavior observed in any patterned surface at high temperature [36].

## Conclusions

In this work, we have studied the formation of nanoholes on GaAs(001) substrates produced after Ga droplet epitaxy at *T*_S_ = 500°C. Our results show that nanodrilling of the GaAs(001) substrate is only possible in the presence of arsenic. We have identified three processes that take place when Ga droplets are exposed to an arsenic flux: (I) local etching by the metallic droplet, (II) GaAs growth by consumption of the Ga droplet under As supplied, and (III) evolution of nanoholes to shallower structures. In this picture, the key role of arsenic flux would be the reactivation of dissolution of the GaAs substrate by the metallic Ga droplets and further GaAs growth, processes that are also in the origin of the well-known flat depressions beneath the Ga droplets in the absence of an arsenic flux. Actuation on the kinetics of the processes involved in nanohole formation may facilitate obtaining nanoholes under design, which ultimately will influence the optical properties of the nanostructures formed inside.

## Competing interests

The authors declare that they have no competing interests.

## Authors’ contributions

All authors carried out the growth of the samples, analysis of the results, and drafted the manuscript. DF carried out the measurements. All authors read and approved the final manuscript.
